# Acute suppurative thyroiditis caused by thyroid papillary carcinoma in the right thyroid lobe of a healthy woman

**DOI:** 10.1186/s13044-018-0049-6

**Published:** 2018-05-15

**Authors:** Hazuki Otani, Masakazu Notsu, Sayo Koike, Miwa Morita, Masahiro Yamamoto, Mika Yamauchi, Takahumi Fuchiwaki, Ichiro Morikura, Noriaki Aoi, Hideyuki Kawauchi, Teruaki Iwabashi, Asuka Araki, Noriyoshi Ishikawa, Riruke Maruyama, Toshitsugu Sugimoto

**Affiliations:** 1 Department of Internal Medicine 1, Shimane University Faculty of Medicine, 89-1 Enya-cho, Izumo, Shimane 693-8501 Japan; 2 Department of Otorhinolaryngology, Shimane University Faculty of Medicine, 89-1 Enya-cho, Izumo, Shimane 693-8501 Japan; 3 Department of Pathology, Shimane University Faculty of Medicine, 89-1 Enya-cho, Izumo, Shimane 693-8501 Japan

**Keywords:** Acute suppurative thyroiditis, Papillary thyroid carcinoma, Fine needle aspiration, Bacterial infection, Piriform sinus fistula, Malignant tumor

## Abstract

**Background:**

The thyroid gland is resistant to microbial infection, because of its organ characteristics such as encapsulation, iodine content, and rich blood supply. Therefore, acute suppurative thyroiditis (AST), as a bacterial infection of the thyroid gland, is rarely seen. AST typically takes places on the left side the neck region in children, because of the coincidence of the left piriform sinus fistula, as a most common route of infection. AST is also usually seen in immunocompromised hosts. Herein, we report a rare case of AST in the right thyroid lobe of adult woman without any immunocompromised condition.

**Case presentation:**

A 59-year-old woman was introduced to our hospital for the further examination with fever, sore throat, and right anterior neck swelling. The patient appeared not to be immunodeficient. Neck ultrasonography showed a 47-mm, hypoechoic, heterogeneous nodule with ill-defined margins and irregular form, suggesting a right thyroid malignant nodule. Fine needle aspiration (FNA) biopsy specimen revealed numerous number of neutrophils in the background without nuclear atypia. Based on the clinical course and cytology, AST was confirmed to be diagnosed. Complete response was obtained by an intravenous administration of antimicrobial agents within a week. Image findings such as CT scan did not show any piriform sinus fistula. Four months later, neck ultrasonography showed a significant decrease in size of the nodule in the right thyroid gland to 27 mm, but the lesion still resembled a malignant nodule. So, FNA was repeated again and cytological examination confirmed papillary thyroid carcinoma (PTC). The patient subsequently underwent total thyroidectomy and bilateral level D1 lymph node dissection. Histological findings revealed a 20-mm PTC in the right lobe with sternothyroid muscle invasion of the tumor.

**Conclusions:**

This report represents a rare case of AST associated with PTC on the right side of thyroid gland, found in a healthy adult woman. The reason why AST coincided with malignant thyroid tumor is unclear. We have to take it into our account that malignant tumor may exist in the background when AST is identified on the right side of thyroid gland with a healthy subject.

## Background

Acute suppurative thyroiditis (AST) is a result of bacterial infection and represents a relatively rare condition in the thyroid gland. The thyroid gland is resistant to microbial infection, because of factors such as its encapsulation, iodine content, and rich blood supply [1, 2]. As a result, AST rarely develops in healthy individuals. Typically, AST is more likely to occur in children and in the left side of the neck. In 80% of patients with AST, the age at onset is before 10 years old (with 30% between birth and 2 years old), and only 8% occur in adulthood [3]. The presence of a left piriform sinus fistula has been reported as important, as a potential route of infection [4]. AST on the right side of thyroid gland in adults is thus rarely seen. We encountered a case of AST in the right lobe of the thyroid in a healthy woman. Moreover, AST developed against a background of papillary thyroid carcinoma (PTC).

## Case presentation

**Fig. 1:**
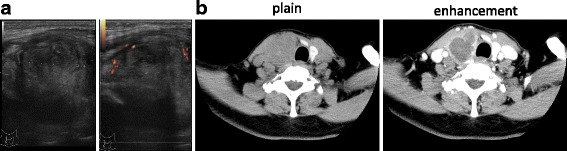
Ultrasonography on day X reveals a hypoechoic lesion with ill-defined margins and irregular form, appearing avascular and heterogeneous (**a**). Computed tomography of the neck on admission (day X + 5) also reveals a low-density lesion in the right thyroid gland, 37 × 37 × 42 mm in size with enhancement in the marginal area (**b**)

**Fig. 2:**
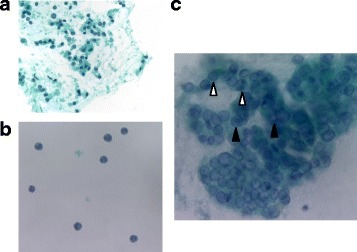
Cytology from FNA shows scant nuclear atypia (**a**), with numerous neutrophils in the background (**b**). Four months later, cytology reveals overlapping cell clusters, high nuclear density, nuclear groove (△), and intranuclear cytoplasmic inclusion bodies (▲), leading to a diagnosis of papillary thyroid carcinoma (**c**)

A 59-year-old woman was introduced to our hospital with a 4-week history of fever, sore throat, and swollen neck, after first visiting a primary-care physician and receiving antibiotics. She had no chronic diseases and appeared not to be to be in an immunocompromised state (she was well-nourished, had no diabetes and did not use any steroids, and human immunodeficiency virus (HIV) antibody was negative). On examination, a nodule showing pain, erythematous changes, and warmth was palpable in the anterior neck on the right side. The nodule showed limited mobility and no adjacent lymphadenopathy. The patient had no medical history of note, and no infectious symptoms such as cough, headache, or abdominal or joint pain were identified, other than the neck pain. Axillary temperature was 36.9°C, heart rate was 109 beats/min, and blood pressure was 169/88 mmHg. Table 1 shows the results of laboratory examination at the first visit (day X). Hematological tests revealed a high erythrocyte sedimentation rate (102 mm/hr) and elevated concentrations of C-reactive protein (10.4 mg/dL). A slight increase in thyroid-stimulating hormone (TSH) was also identified, showing subclinical hypothyroidism. Negative results were obtained for thyroglobulin antibody and serum thyroglobulin level was high (3590 ng/mL). Neck ultrasonography showed a 47-mm nodule, with ill-defined margins, irregular form, and a hypoechoic, heterogeneous appearance, in the area of the right thyroid, suggesting thyroid malignancy (Fig. 1a). Contrast-enhanced computed tomography (CT) confirmed a 37 × 37 × 42-mm nodule within the right thyroid lobe at the middle and lower pole. The thyroid mass resulted in displacement of the trachea toward the left and showed enhancement in the peripheral area of the nodule (Fig. 1b). No signs of metastasis were apparent, including in the lungs and bone. Fine needle aspiration (FNA) was performed on the first visit. Findings on cytological examination were suggestive of AST, because little nuclear atypia was evident, and numerous number of neutrophils were seen in the background (Fig. 2). No PTC was apparent at that time. Based on the clinical course and cytology, AST was confirmed to be diagnosed and the patient was admitted to the endocrinology department.

**Table 1:** Results of laboratory testing at the time of first visit (day X)

WBC	7270	/μL
neutro	67.3	%
eos	0.3	%
baso	0.4	%
mono	6.6	%
lymph	25.4	%
RBC	458 × 10^4^	/μL
Hg	14.2	g/dL
Hct	41.6	%
Plt	24.5 × 10^4^	/μL
ESR	102	mm
Alb	4.2	g/dL
T-Bil	1.0	mg/dL
AST	22	U/L
ALT	17	U/L
γ-GTP	22	U/L
LDH	233	U/L
BUN	9.9	mg/dL
Crea	0.52	mg/dL
CK	71	U/L
CRP	10.4	mg/dL
Na	141	mEq/L
K	4.2	mEq/L
Cl	105	mEq/L
FPG	105	mg/dL
HbA1c	6.1	%
HIV antibody	negative	
Free T3	2.4	pg/mL
Free T4	1.0	ng/dL
TSH	4.55	μU/mL
TPO Ab	< 2.55	IU/mL
Tg Ab	< 6.12	IU/mL
Tg	3590	ng/mL

*ESR* erythrocyte sedimentation rate, *FPG* fasting plasma glucose, *HbA1c* hemoglobin A1c, *TSH* thyroid-stimulating hormone, *Tg* thyroglobulin

**Fig. 3:**
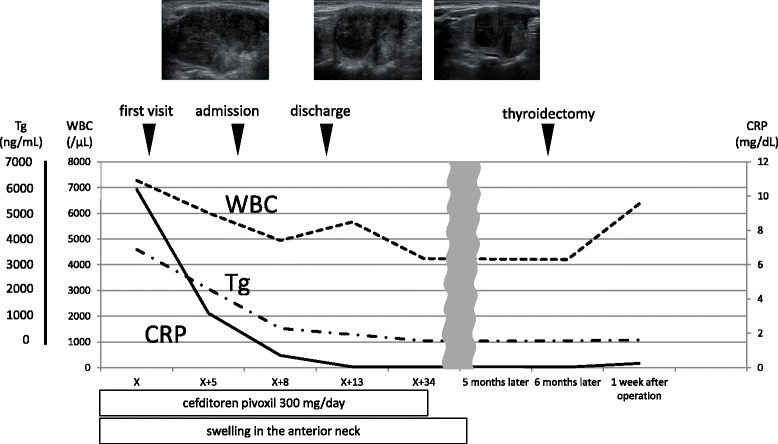
Summary of the clinical course

**Fig. 4:**
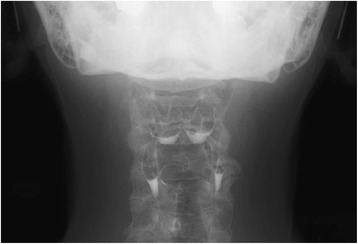
Barium swallow study (frontal view) does not show any fistula from the apex of the pyriform recess

**Fig. 5:**
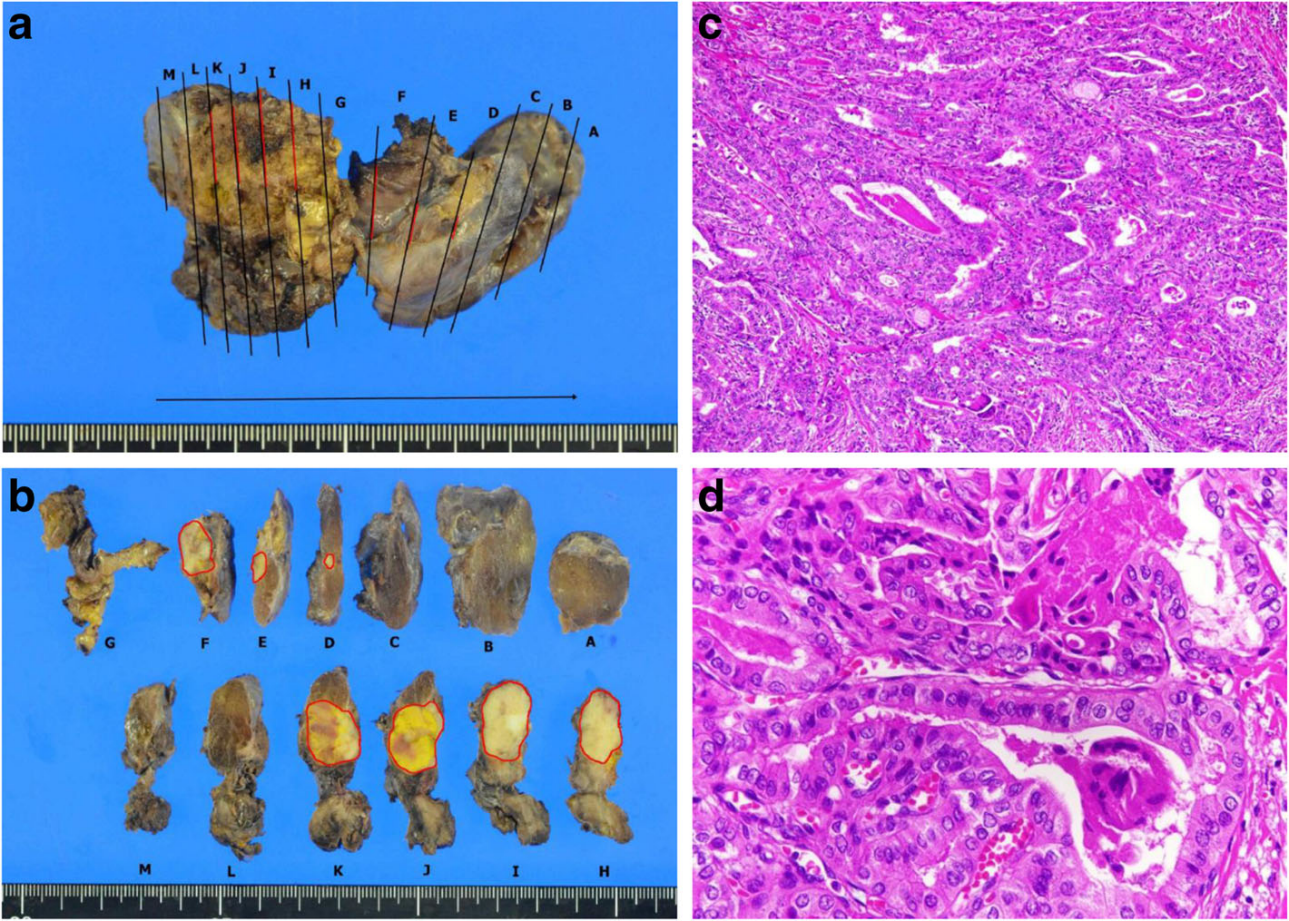
Gross (**a** and **b**) and microscopic (**c** and **d**) appearance of papillary carcinoma of the thyroid

The clinical course is shown in Fig. 3. Antibiotic therapy produced complete response, with rapid improvement within 1 week. Serum thyroglobulin level tended to decrease (528 ng/mL on day X + 8; 47.3 ng/mL on day X + 34). Blood cultures on day X + 6 yielded negative results. CRP concentration was just 0.71 mg/dL on day X + 8, and the patient was discharged with complete disappearance of symptoms. A barium swallow study was performed after CRP turned negative, but no fistula of the pyriform sinus was detected (Fig. 4). Neck ultrasonography 4 months after the onset showed that the nodule in the right thyroid gland had shrunk to 27 mm in diameter, but still showed like malignancy. FNA was repeated, and cytological examination revealed overlapping cell clusters, high nuclear density, nuclear grooves, and intranuclear cytoplasmic inclusion bodies, leading to a diagnosis of PTC (Fig. 2c). The patient subsequently underwent total thyroidectomy and bilateral level D1 lymph node dissection. Histological examination revealed a 20-mm PTC in the right lobe with invasion to the sternothyroid muscle, and a 16-mm PTC in the left lobe (Fig. 5). PTC in the left lobe was not found by ultrasonography before surgery. One possible reason is inhomogenous thyroid grand by adenomatous goiter, but it is unclear why left PTC was not found. Histological examination first detected it after the surgery. The right PTC was well-differentiated, but with large necrotic regions. The left PTC was close to the sternothyroid muscle. No lymph nodes contained metastatic PTC. Cord-like tissue considered as fistulous tract was not found to communicate with the right lobe and the hypopharynx. Pathological staging was pT3N0. Postoperatively, the patient received radioactive iodine ablation.

## Discussion and conclusions

This represents a rare case of right-sided AST concomitant with PTC, found in an adult woman who did not appear immunocompromised and did not have any other foci of infection. In addition, we could not find any evidence of piriform sinus fistula, even after resolution of inflammation.

Some reports have described concomitant AST and thyroid cancer. One case developed AST after FNA of a PTC, and was therefore considered as infection secondary to needle aspiration [5]. Haddad and colleagues reported a case of AST in a patient with ischemic heart failure and type one diabetes mellitus [6]. In another case, a pregnant woman who had given birth by Cesarean section was diagnosed with AST after thyroidectomy [7]. The present case clearly differed from these cases in the lack of a clear cause of AST. The reasons for the AST occurring with malignant tumor in the present case remains unclear, as this woman showed no sign of infectious disease and was not immunocompromised, and no piriform sinus fistula was present. Because the AST and PTC showed identical locations, the PTC could easily be imagined to be infected with bacteria, but the mechanisms were not clear. A previous report [8] suggested that an abnormal blood supply from the PTC could facilitate infection, and an abnormal blood supply from malignant tumor may thus have resulted in infection in our case.

Takai et al. first proposed the presence of a left piriform sinus fistula as a route of infection [4]. In the present case, however, we could not find any sign of a piriform sinus fistula. Repeated inflammation may result in adhesions within the fistula, and potentially masking the fistula in cases with repeated episodes of infection. However, this patient had presented with the first episode of inflammation, so fistula as the route of infection seems unlikely in our case. We attempted to culture bacteria from the thyroid nodule to provide insights into the source of infection, but the results were negative. Cultivation of bacteria probably failed because the patient had been given antibiotics for about 1 week before the sample was obtained for cultivation.

In 80% of AST patients, the age at onset is less than 10 years old, with 30% occurring between birth and 2 years old, and only 8% of cases occur in adulthood [3]. An overview of 109 cases of AST reported that 85 patients had first experienced AST in childhood [9]. Patients diagnosed with AST are likely to have experienced repeated inflammation of the neck, but our patient had no any past history of neck infections. The same overview also reported that 92% of patients showed left-sided infection, with bilateral infection in only 2% [9]. Kingsbury described poor development of the right branchial arch in the prenatal period [10]. Park reported that piriform fistula is more likely on the left than on the right, because the pharyngeal arch is drawn out to the nasal side during formation of the aortic arch by the left fourth branchial arch [11], which is why AST mostly occurs in the left lobe of the thyroid. AST on the right side of thyroid gland in adults is rarely seen. Those cases of right-sided AST in adults that have been reported have shown backgrounds of infection such as infective endocarditis [8, 12] or miliary tuberculosis [13], or immunocompromise due to steroid use or HIV infection [14, 15]. In our case, the patient was HIV-negative and did not have any other infection or underlying diseases and was thus not considered to be in a compromised condition. The cause of AST remains unknown, but the possibility of some involvement of the PTC must be considered.

This case was uncommon in terms of age and lesion location, which made differentiation of AST from malignancy difficult in the early phase. A paper by Lin reviewed 30 patients with malignant thyroid cancer who showed clinical features similar to AST [16]. The significant characteristics of malignant thyroid tumor were clearly indicated as follows: 1) higher age at diagnosis (*P* = 0.0155); 2) presence of dysphonia (*P* = 0.0325); 3) right lobe involvement (*P* = 0.0151); 4) larger thyroid mass (*P* = 0.0013); 5) presence of anemia (*P* = 0.0075); and 6) sterile pus culture (*P* = 0.0013). Our case met 4 of these 6 clinical features suggestive of malignancy. The symptoms and signs of malignant thyroid tumor may mimic those of infectious thyroiditis, so we should be careful in diagnosing AST or aggressive malignant tumor. Long-term follow-up using both ultrasonography and FNA is also necessary. We obtained findings indicating PTC 5 months after identifying the presence of AST, on the third cytological examination. No findings even suggestive of PTC were evident from the first cytological examination, with relatively few variant epithelial cells and numerous leukocytes. One possibility was that we sampled a location where lymphocytes were gathered or that was necrotic.

We encountered a case of AST in the right lobe of a healthy woman. AST can develop with thyroid malignant tumor, so we have to take it into our account that malignant tumor may exist in the background when AST is identified on the right side of thyroid gland with a healthy subject.

## Abbreviations

AST: Acute suppurative thyroiditis
ESR: Erythrocyte sedimentation rate
FNA: Fine needle aspiration
FPG: Fasting plasma glucose
HbA1c: Hemoglobin A1c
PTC: Papillary thyroid carcinoma
Tg: Thyrogloblin
TSH: Thyroid-stimulating hormone
